# Clinical Impact of Sarcopenia in the Decision-Making Process for Patients with Acute Diverticulitis

**DOI:** 10.3390/jcm14010007

**Published:** 2024-12-24

**Authors:** Caterina Puccioni, Pietro Fransvea, Elena Rodolfino, Marco Cintoni, Alessandro Vacca, Dario Benedetto, Maria Cristina Mele, Gabriele Sganga

**Affiliations:** 1 UOC Chirurgia d’Urgenza e del Trauma, Fondazione Policlinico Universitario A. Gemelli IRCCS, 00168 Rome, Italy; caterinapuccioni@libero.it (C.P.); gabriele.sganga@policlinicogemelli.it (G.S.); 2 Università Cattolica del Sacro Cuore, 00168 Rome, Italy; 3 Department of Radiology, Fondazione Policlinico Universitario A. Gemelli IRCCS, Università Cattolica del Sacro Cuore, L.go A. Gemelli, 8, 00168 Rome, Italy; elena.rodolfino@policlinicogemelli.it (E.R.); alessandrovacca1995@gmail.com (A.V.); 4 UOC Nutrizione Clinica, Fondazione Policlinico Universitario A. Gemelli IRCCS, 00168 Rome, Italy; marco.cintoni@guest.policlinicogemelli.it (M.C.); mariacristina.mele@policlinicogemelli.it (M.C.M.); 5 Centro di Ricerca e Formazione in Nutrizione Umana, Università Cattolica del Sacro Cuore, 00168 Rome, Italy; 6 Dipartimento di Matematica, Sapienza Università di Roma, 00168 Roma, Italy; dario.benedetto@uniroma1.it

**Keywords:** sarcopenia, acute diverticulitis, surgical management, outcomes

## Abstract

**Background:** Acute diverticulitis (AD), an inflammatory complication of diverticulosis, affects around 4% of individuals with diverticulosis, with increased incidence in older populations. This study aims to assess the impact of sarcopenia, the age-related loss of muscle mass, on the clinical decision-making and outcomes of patients with AD. **Materials and Methods:** A retrospective study was conducted on 237 patients admitted to the Emergency Department (ED) between January 2014 and February 2022. Patients diagnosed with AD Hinchey ≥ 2 via contrasted tomography (CT) were included. Sarcopenia was assessed using CT scans at the third lumbar vertebra (L3), with skeletal muscle area (SMA) normalized by height to calculate the skeletal muscle index (SMI). Patients were divided into two groups based on sarcopenia status and analysed for surgical outcomes, non-operative management (NOM) success, and complications. **Results:** The prevalence of sarcopenia was 46%. Sarcopenic patients were significantly older and had lower BMI and higher frailty scores. A higher proportion of sarcopenic patients underwent Hartmann, while non-sarcopenic patients more often had anastomosis. Sarcopenia did not significantly affect overall morbidity, mortality, or the failure rate of NOM. However, sarcopenic patients with a BMI > 25 had a higher likelihood of requiring redo surgeries. **Conclusions:** Sarcopenia plays a critical role in the surgical management of AD but does not predict worse clinical outcomes. The decision to perform surgery, particularly Hartmann’s procedure, is influenced by sarcopenia, yet morbidity and mortality rates are comparable between sarcopenic and non-sarcopenic patients. These findings highlight the need for sarcopenia to be considered in preoperative assessments.

## 1. Background

Colonic diverticulosis is defined as the presence of diverticula, which are bulging pockets that push through weak spots in the colonic wall. The prevalence of diverticulosis in individuals over 40 years old is about 20%, increasing to 60% in those over 60 years old [1]. Risk factors for the development of diverticula include age, male gender, smoking, and overweight [1,2]. Acute diverticulitis (AD), on the other hand, is defined as the inflammation of one or more diverticula. Approximately 4% of patients with diverticulosis will experience diverticulitis at least once in their lifetime, and this risk increases with age [3]. The average age of patients presenting to the Emergency Department (ED) with diverticulitis is 63 [4]. Sixteen percent of patients with diverticulitis are younger than 45 years old, highlighting the significance of diverticulitis among younger individuals as well [5]. Numerous classifications exist to distinguish the stages of the disease; one of the most commonly used is the Hinchey Classification, which divides Acute Diverticulitis (AD) into four stages based on the degree of intra-abdominal contamination [6]. While conservative treatment often manages AD successfully, a notable number of patients still require surgical intervention. Surgical approaches vary and it is possible to either reconstruct the intestinal tract with an anastomosis or perform a stoma to protect or avoid the anastomosis [7,8,9]. In particular, it is possible to perform a Hartmann’s procedure, which consists of the removal of part of the colon and rectum, sealing the remaining rectum and redirecting the remaining colon to a colostomy. Otherwise, it is possible to perform a sigmoidectomy removing part of the left colon and joining the two remnant parts of the colon creating an anastomosis. Another possibility for the surgeon is to perform a laparoscopy with just a lavage and a drainage of the abscess, less used but sometimes a feasible alternative to a colonic resection [9]. For surgeons, predicting the risk of complications in each patient is crucial to choose the most valid surgical approach, considering various risk factors, clinical features, and comorbidities present in different individuals [10,11,12,13,14,15]. Among the several patient characteristics, classifications, and indexes available in the literature that aim to serve as preoperative predictors of adverse perioperative events, sarcopenia is gaining increasing attention. First described by Rosenberg in 1989, sarcopenia is defined as the loss of skeletal muscle mass and strength associated with aging. Studies indicate a loss of skeletal muscle mass of 6% every 10 years after the age of 50, 14% between the age of 65 and 70, and up to 53% beyond the age of 80, with a prevalence reaching 29% in the elderly and ranging from 11% to 50% in those over 80. Thus, sarcopenia cannot be considered exclusively a condition of the elderly but can appear at any age [16,17,18,19,20,21,22,23,24,25,26,27,28,29,30]. The purpose of this study is to analyze the clinical impact of sarcopenia on the decision-making process for patients with acute diverticulitis.

## 2. Materials and Methods

### 2.1. Study Design

A retrospective study was conducted enrolling patients admitted to the Emergency Department (ED) with a diagnosis of Acute Diverticulitis (AD) between 1 January 2014 and 28 February 2022. Inclusion criteria were age > 18 years old, contrasted tomography (CT) diagnosis of AD with a Hinchey stage ≥ 2, no other inflammatory/infective diseases, and patient’s capability to give consent. Exclusion criteria were a history of Chronic Obstructive Pulmonary Disease (COPD), asthma or other interstitial pulmonary diseases, absence of abdominal CT scan, oncological patients or patients under corticosteroids/immunosuppressive therapies, pregnancy, and patients not capable of giving consent.

### 2.2. Sarcopenia Measurement

Our radiologist used a specific software (the Slice-O-Matic software, version 6 Tomovision, Montreal, QC, Canada) to analyze the CT images in order to assess the sarcopenia. Single CT slices of the third lumbar vertebrae were identified as the standard landmark. Each slice was anatomically localized using coronal and sagittal multiplanar reformats (MPRs) to ensure it specifically lies at the third lumbar vertebra (L3). Slices were analyzed as Digital Imaging and Communication in Medicine (DICOM) images obtained from a picture archiving and communication system (PACS). To measure the skeletal muscle area (SMA), the following Hounsfield Unit (HU) thresholds were used: −29 to +150 for skeletal muscle area (SMA), −190 to −30 for intermuscular adipose tissue (IMAT), −150 to −50 for visceral adipose tissue (VAT), and −190 to −30 for subcutaneous adipose tissue (SAT). Our radiologists did not know either the outcome of the CT scans or the patient’s need for surgery. In a team with radiologists and nutritionists, we normalized the SMA value by the height of each patient, obtaining the skeletal muscle index (SMI, cm^2^/m^2^). SMI, according to the literature, is a more accurate predictor of the entire muscle area of the patient. As for other studies, we used a cut-off value of an SMI of 44.9 cm^2^/m^2^ to distinguish between sarcopenic patients and non-sarcopenic ones [7,8].

### 2.3. Scan Acquisition

CT scans were performed by using a multi-detector row helical scanner (Siemens—Somatom Definition Flash, Munich, Germany). CT images were obtained from the level of the diaphragm to the symphysis pubis with 128 × 0.6 mm-section collimation, 0.5 s gantry rotation, pitch of 6, and 1.00 mm slice thickness. CT-enhanced images were acquired 70 s (portal phase) after a bolus intravenous injection of iodinated non-ionic contrast medium (Iopromide 370 mgI/mL, Ultravist; Bayer Schering Pharma, Berlin, Germany), at a flow rate of 3 mL/s via an antecubital vein. To minimize variability in the enhancement of the abdominal organs related to differences in patient size, the volume of contrast material was calculated according to the body weight of the patient (2 mL of contrast material per kilogram of body weight). The ensuring average volume of contrast material was equal to 130 mL (range, 120–140 mL).

### 2.4. Variables and Statistical Analysis

Variables taken into account were personal data (age, gender), physio pathologic variables (body mass index—BMI, blood pressure—BP, heart rate—HR, shock index—SI), laboratories variables (hemoglobin—Hb, white blood cells—WBC, platelets, sodium, potassium, chloride, lactate dehydrogenase, gamma glutamyl transferase, transaminase, creatinine, blood sugar), comorbidities data (using the America Society of Anesthesiologists (ASA) score, the Charlson Comorbidity Index (CCI) and the Frailty Index), and outcomes variables (imaging, surgery, postoperative period, 30-day mortality). Postoperative complications were classified using the Clavien–Dindo classification [31]. Considering the presence or the absence of sarcopenia at arrival in the ED, patients were divided into two groups: Group1_sarcopenia and Group2_control. The two groups were compared analyzing the relationship between sarcopenia and postoperative mortality or complications and the relationship between sarcopenia and the need for surgery. The study was conducted according to the Declaration of Helsinki for Biomedical Research. Due to the retrospective and anonymous nature of the study based on observational only analysis, with no influence on the choice of treatment, an Ethics Committee vote was not necessary.

### 2.5. Statistical Analysis

Statistical analysis was carried out using SPSS version 16.0 for MacOsX. Dichotomous data and counts were presented in frequencies, whereas continuous data were presented as mean values  ±  standard deviations (SD) and/or median with 25–75 interquartile range (IQR) and minimum–maximum range. Differences between means were compared using the Mann–Whitney U test or the Kruskal–Wallis test when indicated. Pearson χ^2^ test, with or without Yates correction, was used to compare the differences in frequencies; t-student test, Pearson test, and ANOVA test were used when needed. A descriptive analysis was used to illustrate the patient population, with continuous variables summarized by median and interquartile range and categorical variables presented with percentages. In addition to the univariate analysis, a multivariate analysis was also performed, using a linear logistic regression model considering sarcopenia as the dependent variable. Values of *p* < 0.05 were considered significant. To test the accuracy of sarcopenia in predicting postoperative complications and the need for surgery, we used at first the Kolmogorov test to assess the presence or absence of sarcopenia. If present, a logistic regression was conducted between the independent variable (sarcopenia, categorical) and the dependent variable (presence of complications, categorical). If absent, the evaluation was performed using non-parametric tests, in particular the rank test.

## 3. Results

Between the 1 January 2014 and 28 February 2022, we enrolled 315 patients. Of them, 78 were excluded from the analysis because they did not fit the inclusion criteria. Our final study population included 237 patients. Mean age was 61 ± 15.6 years (45–77), with a small prevalence of male (132, 55.7% vs. 105, 44.3% female patients). Among the 237 patients analyzed, 173 (73.0%) were treated at presentation to the ED with non-operative management (NOM). In total, 116 patients (49%) underwent surgery: in 64 (55%) of them, the surgery was the first choice of treatment because of the patients’ clinical, laboratories, and radiological conditions; in 52 (45%) of them, the surgery was carried out due to the failure of the NOM approach. The failure of the NOM rate was 30% (52 patients). The mean length of stay (LOS) was 9.8 ± 9.8 days, with a mortality rate of 5% (12 patients). Moreover, the complication rate was 26.6% (62 patients) with a distribution according to the Clavien–Dindo Classification (CD) as follows: 32.2% with CD 1, 24.2% with CD 2, 22.6% with CD 3a, 12.9% with CD 3b, 3.2% with CD 4a, and 4.9% with CD 4b. Considering the distribution of our population according to Hinchey stage, we found that patients with Hinchey > 4 underwent surgery as a first choice of treatment as suggested by the latest guidelines. Patients with Hinchey 2b to 4 were homogeneous in terms of treatment options and choices. These patients are also the ones for whom the guidelines are vaguer, not strongly suggesting NOM over surgery, as numerous patient’s characteristics have to be taken into account. All the variables analyzed were reported in Table 1, distinguished among patients treated with NOM or surgery. The ones undergoing surgery were older (surgical: 63.7 y/o ± 15 vs. NOM: 58.4 y/o ± 16, *p* < 0.009), with a lower BMI (surgical: 25 ± 3.7 vs. NOM: 26.4 ± 5, *p* < 0.01), a higher creatinine (surgical: 1.43 mg/dL ± 1.26 vs. NOM: 0.89 mg/dL ± 0.26, *p* < 0.0001), and urea (surgical: 22.8 mg/dL ± 21.1 vs. NOM: 17.6 mg/dL ± 14.6, *p* < 0.03) values and a lower albumin value (surgical: 29.8 g/L ± 7 vs. NOM: 35.4 g/L ± 5.2, *p* < 0.0001) at the arrival at the ED. For patients undergoing surgery, the median time from ED admission to intervention is 1 with a mean of 3 (±5). Furthermore, 52 patients (44.8%) among the surgical group underwent surgery after a failed attempt of NOM. Analyzing the surgical group, surgical patients had a longer LOS (surgical: 14.2 ± 12 days vs. NOM: 5.6 ± 3.6 days, *p* < 0.0001) and a higher morbidity (surgical: 52.6%—61 patients vs. NOM: 16%—28 patients) and mortality (surgical: 8.6%—10 patients vs. NOM: 1.6%—2 patients) rate. In Table 2, we compared all the variables between the two most common surgeries performed: Hartmann’s procedure (64 patients, 55.2% of surgical patients) versus colon resection with anastomosis (37 patients, 31.9% of surgical patients). As expected, patients undergoing Hartmann’s procedure were older (Hartmann: 68.5 y/o ± 12.3 vs. Anastomosis: 54.5 y/o ± 12.6, *p* < 0.001), with a higher grade of Hinchey (Hartmann’s Hinchey > 3: 50% vs. Anastomosis’s Hinchey > 3: 13.5%, *p* < 0.002), and a higher Charlson Comorbidity Index (CCI) (Hartmann: 3.4 ± 2.6 vs. Anastomosis: 1.1 ± 1.6, *p* < 0.001) and Frailty Index (Hartmann: 2.25 ± 1.25 vs. Anastomosis: 1.48 ± 0.96, *p* < 0.001). Moreover, patients undergoing Hartmann’s Procedure experienced a higher complication rate (33 patients—51.5%) than patients undergoing resection and anastomosis (nine patients—24.3%, *p* < 0.07), even though the mortality rate was not statistically significant (Hartmann: six patients—9.4% vs. Anastomosis: two patients—5.4%, *p* = 0.47). The comparison between patients successfully treated with NOM versus patients in which NOM failed is reported in Table 3. There was no statistically significant difference in terms of mean age (Success NOM: 58.4 ± 15.9 vs. Failed NOM: 59.7 ± 14.6, *p* = 0.61) and sex (Success NOM: 68 male—56.2% vs. Failed NOM: 28 male—54%, *p* = 0.77). The Hinchey grade (Success NOM-Hinchey ≥ 3: four patients—3.3% vs. Failed NOM-Hinchey ≥ 3: 4 pts—7.7%, *p* = 0.2), CCI (Success NOM: 1.8 ± 2.1 vs. Failed NOM: 2.4 ± 2.6, *p* = 0.11), and Frailty Index (Success NOM: 1.5 ± 0.8 vs. Failed NOM: 1.5 ± 0.9, *p* = 1) were also not statistically significant. Instead, we found a significative statistical difference between morbidity (Success NOM: zero pts—0% vs. Failed NOM: 28 pts—53.8%, *p* < 0.001) and mortality rate (Success NOM: two patients—1.6% vs. Failed NOM: four patients—7.7%, *p* < 0.05). Analyzing the morbidities, patients treated with NOM successfully did not experience specific complications, unlike patients who failed NOM who experienced typical complications of surgical procedures.

Moreover, we conducted further analysis dividing our population sample into three groups according to the SMI value. We identified groups named Low SMI (SMI ≤ 35), Intermediate SMI (SMI 35–45), and Normal SMI (SMI > 45).

Evaluating these three groups (Table 4, we found that patients in the Low SMI group are older (Low 72 (±12.7), Intermediate 63 (±14.5), Normal 57 (±15.5), *p* < 0.001), frailer (Frailty Index: Low 2.2 (±1.2), Intermediate 1.6 (±0.8), Normal 1.5 (±0.8), *p* < 0.001), with more comorbidities (CCI: Low 3.6 (±2.7), Intermediate 2.3 (±2.2), Normal 1.9 (±2.5), *p* 0.004). Additionally, the number of female patients is higher in the Low and Intermediate SMI groups than in the Normal one (Low: 19 female patients (61%), Intermediate: 49 female patients (62%), Normal: 37 female patients (29%), *p* < 0.001), confirming that women are more subject to sarcopenia than men, as the literature already suggests.

Continuing our analysis, we did not find any statistic difference among the three groups in terms of Hinchey grade, as if the severity of the diverticulitis is well distributed in our samples (*p* = 0.6).

What is interesting is that although patients with low SMI undergo surgery more often than those with normal SMI (Low: 20 surgical patients (64.5%), Intermediate: 42 surgical patients (53.2%), Normal: 54 surgical patients (42.5%), *p* = 0.059), there is no difference in terms of failure of NOM (Low: 7 NOM failure (38.9%), Intermediate: 19 NOM failure (33.9%), Normal: 26 NOM failure (26.3%), *p* = 0.83). At the same time, we found a statistically significant difference in terms of type of surgery, with a Hartmann’s procedure more often used in patients with Low SMI (Low: 16 Hartmann’s (80%), Intermediate: 24 Hartmann’s (57.1%), Normal: 24 Hartmann’s (44.4%), <0.05) and a higher mortality rate (Low: four deceased patients (12.9%), Intermediate: zero deceased patients (0%), Normal: eight deceased patients (6.3%), *p* = 0.014).

Moreover, our study spans a period from 2014 to 2022. In fact, in 2020, the WSES published an update of the guidelines for the management of acute colonic diverticulitis in the emergency setting. For that reason, we try to analyze our data dividing the sample into two more groups: those treated before the 2020 guidelines update and those treated after. We did not find any statistically significant difference among the choice between NOM or surgical treatment, nor among the NOM failure rate or the type of surgical procedure (Table 5). It is possible to highlight that, although not statistically significant, before 2020, we used surgical procedures different from Hartmann’s resection or sigmoidectomy with anastomosis, such us lavage and drainage or resection with ileostomy, in 15.3% of patients, instead of after 2020 where we used other procedures in only 5.6% of patients.

### Impact of Sarcopenia in Our Population

The prevalence of sarcopenia in our general population (237 patients) was 46% (109 patients). Thus, we divided our patients into two groups: Sarcopenic (109 patients, 46%) and Not Sarcopenic (128 patients, 54%) (Table 6). From our analysis, it emerged that the Sarcopenic population was significantly older (mean age Sarcopenic 66 y/o ± 14.4 vs. Not Sarcopenic 56.7 y/o ± 15.5, *p* < 0.0001), with a significant prevalence of female sex (Sarcopenic: 68 female—62.4% vs. Not Sarcopenic: 37 female—29% *p* < 0.001) and a lower BMI (Sarcopenic: 24.7 ± 3.9 vs. Not Sarcopenic: 26.5 ± 4.7, *p* < 0.002). Considering the stage of diverticulitis at arrival, we did not highlight any difference between the two groups in terms of Hinchey 2 (Sarcopenic: 86 patients—78.9% vs. Not Sarcopenic: 104 patients—81.3%, *p* = 0.65), Hinchey 3 (Sarcopenic: 10 patients—9.2% vs. Not Sarcopenic: 11 patients—8.6%, *p* = 0.87), Hinchey 4 (Sarcopenic: 13 patients—11.9% vs. Not Sarcopenic: 13 patients—10.2%, *p* = 0.66). Moreover, we found a statistical difference in terms of Charlson Comorbidity Score (CCS) (Sarcopenic: 2.6 ± 2.4 vs. Not Sarcopenic: 1.9 ± 2.5, *p* < 0.03) and Frailty Index (Sarcopenic: 2.4 ± 0.87 vs. Not Sarcopenic: 1.8 ± 0.76, *p* < 0.0001) at the univariate analysis that was not confirmed at the multivariate. Sarcopenia prevalence in our 173 patients who underwent NOM was 42.2% (73 patients), in contrast with the sarcopenia prevalence of 53.4% (62 patients) among patients who underwent surgery, with *p* < 0.056. The failure of NOM was observed among our sarcopenic patients more than among the non-sarcopenic ones (Failure NOM Sarcopenic: 26 patients—35.6% vs. Failure NOM Not Sarcopenic: 26—26% *p* > 0.17), with a slightly higher mortality rate in the first group (Sarcopenic: eight patients—6.2%—vs Not Sarcopenic: four patients—3.7%, *p* > 0.5) (Table 6). After that, we analyzed surgical population (Table 7) highlighting how sarcopenic patients more often underwent a Hartmann’s procedure than non-sarcopenic ones (Sarcopenic: 40 patients—64.5% vs. Not Sarcopenic 24 patients—44.4%, *p* < 0.03). At the same time, in the Not Sarcopenic group, the anastomosis was performed more frequently (Not Sarcopenic 23 patients—42.6% vs. Sarcopenic 14 patients—22.6%, *p* < 0.02). Considering the overall morbidity rate, we had 47% (55 patients) of our patients who experienced a complication and of them 60% had a Clavien–Dindo ≥ 2. Among patients who underwent a Hartmann’s procedure, we found that sarcopenic patients had a higher morbidity, although not statistically significant (Not Sarcopenic 17 patients vs. Sarcopenic 21 patients, *p* > 0.14). This *p* is also confirmed if we divide our morbidity rate among different Clavien–Dindo classes (*p* = 0.17). In parallel, we did not highlight a difference in terms of length of stay (Sarcopenic: 13.5 ± 9.3 days, vs. Not Sarcopenic: 16.4 ± 16.2 days, *p* > 0.36), nor in the mortality rate (Sarcopenic eight patients—20% vs. Not Sarcopenic three patients—12.5%, *p* = 0.44) (Table 8). When investigating patients who underwent anastomosis (Table 8), we did not find any statistically significant difference in terms of morbidity (Sarcopenic: four patients—28.6%, vs. Not Sarcopenic: seven patients—30.4%, *p* = 0.55) and mortality (Sarcopenic: zero patients—0% vs. Not Sarcopenic: two patients—8.7%, *p* = 0.57). If we stratify our surgical population according to the Hinchey class, we find a statistically significant difference considering Clavien–Dindo < 3 (*p* < 0.03), not confirmed considering morbidity > 3 and mortality. In our multivariate analysis, if sarcopenia is associated to being overweight (BMI > 25), regardless of the Hinchey class, there is a higher chance for the patient to need a redo surgery (*p* < 0.05).

## 4. Discussion

In recent years, the interest in optimizing surgical patients to minimize morbidity and mortality has grown significantly. However, emergency surgeons face the dual challenges of treating increasingly elderly and frail patients and the inability to implement optimization phases. Therefore, in the emergency setting, it is crucial to identify non-modifiable factors that can guide the surgeon in making the best therapeutic decisions [32,33,34,35,36]. Sarcopenia, defined as the loss of muscle mass and strength, has a substantial social impact due to its role in increasing the risk of falls and fractures [17]. It also negatively affects the ability to perform daily activities and is associated with cardiovascular, respiratory, and cognitive diseases, leading to motor difficulties [18]. These effects significantly impact healthcare costs by increasing the risk of hospitalization and the need for more complex treatments. Numerous studies have shown how sarcopenia influences the length of hospital stay and the associated costs [21,22]. For instance, a study in the Czech Republic found that sarcopenic elderly patients cost twice as much as their non-sarcopenic counterparts [23]. Similarly, a Portuguese study demonstrated that the cost of sarcopenic patients is higher regardless of age. In the United States in 2000, the estimated direct healthcare cost attributable to sarcopenia was USD 27 billion [24]. Several strategies have been proposed by the International Clinical Practice Guidelines for Sarcopenia (ICFSR) in 2018 and the SPRINT project consortium in 2022 to prevent and reduce the development of sarcopenia, including resistance exercises involving skeletal muscle contraction using external resistances such as weights, bands, and body weight [25,26]. While primary sarcopenia is related to aging with no identifiable causes, and secondary sarcopenia is multifactorial, it appears that both contribute to muscle loss in elderly patients. This muscle loss is often accompanied by an increase in fat mass, resulting in a relatively stable body weight—a condition known as sarcopenic obesity, increasingly observed in the elderly population. A cross-sectional study of 2.208 subjects aged 55 years or older demonstrated that reduced strength (measured by hand grip strength) and walking limitations (less than 1.2 m/s or difficulty walking 400 m in under 15 min) are correlated with increased body fat [27,28]. The prevalence of walking limitations is significantly higher in patients with high body fat percentage and low hand grip strength (61%) compared to those with high hand grip strength and low body fat percentage (7%). Furthermore, studies have highlighted the correlation between inflammatory status and sarcopenia, showing a positive relationship between levels of C-reactive protein, interleukin-6, and sarcopenia. Recent studies have indicated that sarcopenia plays a role in surgical outcomes. Sarcopenic patients undergoing major elective surgery are at a greater risk of complications compared to non-sarcopenic patients [29,30,37,38,39,40,41]. Our study aimed to evaluate sarcopenia as a potential factor influencing surgical decisions in an emergency setting, not just in elective procedures where preoperative risk factors are assessed. In accordance with the literature, our study found a statistically significant correlation between the patient’s age and sarcopenic status, as well as between BMI and sarcopenia [42,43]. Our sarcopenic group had a higher mean age (66.5 years), supporting the concept that muscle mass loss is directly proportional to aging. Additionally, we observed a positive correlation between sarcopenic status and proposed surgical procedure, with a higher rate of Hartmann’s procedures among patients with reduced muscle mass, compared to a higher frequency of colorectal anastomoses in better-nourished patients. This likely reflects the surgeon’s confidence that well-nourished patients’ bowels heal more quickly and safely, although this belief is not supported by evidence [44,45]. In fact, analyzing outcomes based on the type of surgery, we found no significant differences in minor and major complications or mortality rates between sarcopenic and non-sarcopenic groups, given disease severity homogeneity. In particular, our study highlights how sarcopenia is not associated with the worst surgical outcomes, intended as postoperative morbidity defined with the Clavien–Dindo classification (Table 7). At the same time, sarcopenic patients are not at higher risk of anastomotic leak, allowing the surgeon to perform a resection with anastomosis regardless of the sarcopenic status of the patient. This is fundamental considering that sarcopenic patients undergo Hartmann’s procedures more often than anastomosis, probably because of a surgeon’s prejudice during the evaluation process. Dividing our sample into three groups according to the SMI, patients in the “Low SMI” group had a higher statistically significant mortality rate (Table 4) despite not having a significant difference in terms of other postoperative complications (morbidity).

In order to further investigate the potential role of sarcopenia in postoperative complications, we developed a logistic regression model including several variables (sarcopenia, age, sex, BMI, frailty, Charlson Comorbidity index). The results from the analysis, as shown in Table 9, indicate that none of the variables analyzed achieved statistical significance in predicting the occurrence of postoperative complications. Specifically, the *p*-values for all variables, including sarcopenia, were above the conventional threshold for significance (*p* > 0.05).

Although the odds ratio (OR) for sarcopenia was 1.13, suggesting a potential association with postoperative complications, the high *p*-value (0.78) and the wide confidence intervals (ranging from 0.49 to 2.56) indicate a lack of statistical support for a meaningful relationship. This suggests that while sarcopenia may appear to have a positive association with postoperative complications in our data, this effect is not strong enough to be considered reliable or consistent across the sample.

The absence of significant findings in this analysis could be due to several factors. First, the sample size may have been insufficient to detect small to moderate effects, particularly if the relationship between sarcopenia and postoperative morbidity is subtle. Additionally, the variability in the variables themselves (e.g., BMI, frailty score) or the potential presence of unmeasured confounding factors might have diluted the observed effects. Finally, it is also possible that sarcopenia does not have a direct or independent impact on postoperative complications in this specific population, and its role could be more complex, potentially interacting with other factors not fully captured in this model.

Therefore, while our findings do not support a statistically significant association between sarcopenia and postoperative complications, further studies with larger sample sizes and more granular data on sarcopenia, frailty, and other relevant clinical factors are needed to better understand the potential relationship. At the same time, while our analysis did not find a significant association between sarcopenia and postoperative complications, this negative finding is still important for clinical decision-making. Specifically, the lack of a significant relationship between sarcopenia and complications suggests that sarcopenia may not need to be a determining factor when considering surgical approaches for patients with diverticulitis. In other words, surgeons may not need to adjust their choice of surgical procedure based solely on the presence of sarcopenia in these patients, as it does not appear to significantly affect postoperative outcomes in this cohort.

This finding could have important implications for the management of surgical patients, particularly in avoiding overemphasis on sarcopenia as a risk factor, in particular in anastomotic leak and other feared postoperative complications.

Few studies have examined the impact of sarcopenia in elective surgery, and even fewer have explored the role of nutritional status in emergency settings and diverticulitis. K. Matsushima et al. demonstrated a statistically significant correlation between sarcopenia and outcomes in a sample of 89 patients undergoing surgery for acute diverticulitis between 2007 and 2014, measuring the area and transverse diameter of the psoas muscle at the L3 level, not normalized for height. They observed that patients with less muscle at the L3 level, defined as sarcopenic, experienced more complications. G. Simpson et al. later showed that psoas muscle mass at the L3 level, normalized for height, correlates with patient outcomes, with lower values associated with higher complication rates [19]. Both studies used the psoas muscle volume at the L3 level, though the literature suggests that the skeletal muscle index (SMI) may be a better parameter for defining sarcopenic status and correlating with clinical outcomes. More recently, D. Schaffler-Schaden et al. analyzed 99 patients who underwent surgery for diverticulitis between 2012 and 2018, distinguishing sarcopenic and non-sarcopenic patients according to the skeletal muscle index and using literature-defined cut-offs [20]. Similar to our study, the Salzburg group did not show statistically significant differences in outcomes. Thus, considering our study, the surgeon’s preoperative decision-making process is likely influenced by the patient’s nutritional status, though this does not correlate with morbidity rates in our sample. Furthermore, our data suggest that the decision between anastomosis and stoma should be made independently of sarcopenic status, considering the recanalization rate.

## 5. Limitations

Conducing our study, we focused on some possible bias. The study’s retrospective single-center nature limits the ability to establish causality between sarcopenia and outcomes in acute diverticulitis. Differences in patient populations, clinical practices, and resource availability in other settings might influence the outcomes. Measurement of sarcopenia: The use of the SliceOMatic software to analyze skeletal muscle area (SMA) at the level of the third lumbar vertebra, normalized to height to obtain the skeletal muscle index (SMI), may not capture the full complexity of sarcopenia. Variability in measurement techniques and cut-off values for SMI may affect the classification of sarcopenic status. Although multivariate analysis was performed, residual confounding factors may still exist. Variables such as nutritional status, physical activity, and other comorbid conditions were not fully controlled, which might influence outcomes. Limited outcome measures: The study primarily focuses on sarcopenia’s impact on surgical outcomes and complications. Other important aspects, such as long-term functional status, quality of life, and readmission rates, were not assessed. Due to the retrospective and observational nature of the study, an ethics committee vote was deemed unnecessary. However, this might limit the depth of patient consent and involvement in the research process.

Study period: In our study, we considered a long study period, from 2014 to 2022. In fact, in 2020, the WSES published an update of the guidelines for the management of acute colonic diverticulitis in the emergency setting. For that reason, we try to analyze our data dividing the sample into two more groups: those treated before the 2020 guidelines update and those treated after. Large range of SMI values: Another selection bias could be that patients with more severe sarcopenia may be more likely to undergo surgery or to require longer hospitalization. For that reason, dividing our sample in three groups according to SMI values, we tried to overcome this selection bias and in particular we did not find any statistically significant difference in terms of LOS, NOM failure, morbidities and anastomotic leak.

Addressing these limitations in future research could help to better understand the relationship between sarcopenia and outcomes in acute diverticulitis, enhancing the evidence base for clinical decision-making in this context.

## 6. Conclusions

In conclusion, this retrospective study underscores the significant impact of sarcopenia on the clinical outcomes of patients with acute diverticulitis. Our findings reveal that sarcopenic patients, who tend to be older and have a lower BMI, are more likely to undergo a Hartmann’s procedure compared to non-sarcopenic patients. Despite this, the overall complication and mortality rates did not differ significantly between sarcopenic and non-sarcopenic groups, suggesting that while sarcopenia influences surgical decision-making, it does not necessarily predict worse outcomes. Additionally, the failure rate of non-operative management (NOM) was not significantly correlated with sarcopenic status, indicating that NOM can be considered regardless of a patient’s nutritional state. These results emphasize the importance of considering sarcopenia in the preoperative assessment and decision-making process, but also highlight the need for further research to explore its implications in emergency surgery.

Overall, this study adds to the growing body of evidence suggesting that while sarcopenia is a crucial factor in determining surgical approaches, it does not solely dictate patient prognosis. Future studies should focus on developing comprehensive management strategies that incorporate both nutritional status and other patient-specific factors to optimize outcomes in acute diverticulitis.

## Figures and Tables

**Table 1:** Overall population analysis: Non-operative management vs. surgery.

Variables	General Population (237)	Surgical (116)	NOM (173)	*p*
Age (y/o)	61 (±15.6)	63.7 (±15)	58.4 (±16)	0.009
Gender				
Male	132 (55.7%)	64 (54.7%)	68 (56.2%)	0.81
Female	105 (44.3%)	53 (45.3%)	53 (43.8%)	
BMI	24.9 (±3.9)	25 (±3.7)	26.4 (±5)	0.01
Frailty Index	1.61 (±0.92)	1.9 (±1.1)	1.5 (±0.8)	0.004
CCI	2.3 (±2.5)	3 (±2.8)	2 (±2.3)	0.001
LOS	9.8 (±9.8)	14.2 (±12)	5.6 (±3.6)	0.0001
SMI	47.9 (±24.8)	50 (±28.1)	42.3 (±9.6)	0.001
SMI < 44.9 cm^2^/m^2^	109 (46%)	62 (53%)	47 (39%)	0.02
Creatinine (mg/dL)	1.15 (±1.24)	1.43(±1.26)	0.89 (±0.26)	0.0001
Urea (mg/dL)	20.2 (±18.3)	22.8 (±21.1)	17.6 (±14.6)	0.03
Albumin (g/L)	32.4 (±6.8)	29.8 (±7)	35.4 (±5.2)	0.0001
Hinchey				
2	190 (80.2%)	73 (62.9%)	164 (94.8%)	0.001
3	21 (8.8%)	17 (14.7%)	9 (5.2%)	0.005
4	26 (11%)	26 (22.4%)	0 (0%)	0.001
Morbidity	62 (26.2%)	61 (52.6%)	28 (16%)	0.001
Mortality	12 (5%)	10 (8.6%)	2 (1.6%)	0.01

BMI: body mass index; CCI: Charlson Comorbidity index; LOS: length of stay; SMI: skeletal muscle index.

**Table 2:** Overall comparison of Hartmann’s Procedure and Colon Resection with Anastomosis.

Variables	Hartmann’s Procedure (64)	Resection + Anastomosis (37)	*p*
Age (y/o)	68.5 (±12.3)	54.5 (±12.6)	0.001
Gender			
Male	32 (50%)	27 (73%)	0.02
Female	32 (50%)	10 (27%)	
BMI	25.1 (±3.9)	24.9 (±3.5)	0.79
Hinchey ≥ 3	32 (50%)	5 (13.5%)	0.002
SMI < 44.9 cm^2^/m^2^	40 (62.5%)	14 (37.8%)	0.016
CCI	3.4 (±2.6)	1.1 (±1.6)	0.001
Frailty Index	2.25 (±1.25)	1.48 (±0.96)	0.001
LOS	14.5 (±12.3)	11.7 (±9.1)	0.23
Morbidity	33 (51.5%)	9 (24.3%)	0.007
Mortality	6 (9.4%)	2 (5.4%)	0.47

SMI: skeletal muscle index BMI: body mass index; Charlson Comorbidity index; LOS: length of stay.

**Table 3:** Comparison between patients successfully treated with NOM versus NOM failure.

Variables	Successful NOM (121)	Failed NOM (52)	*p*
Age (y/o)	58.4 (±15.9)	59.7 (±14.6)	0.61
Gender			
Male	68 (56.2%)	28 (54%)	0.77
Female	53 (43.8%)	24 (46%)	
BMI	26.3 (±5)	24.7 (±3.5)	0.04
Hinchey ≥ 3	4 (3.3%)	4 (7.7%)	0.2
SMI (cm^2^/m^2^)	51.7 (±32.9)	46 (±9.8)	0.22
SMI < 44.9 cm^2^/m^2^	47 (38.8%)	26 (50%)	0.17
CCI	1.8 (±2.1)	2.4 (±2.6)	0.11
Frailty Index	1.5 (±0.8)	1.5 (±0.9)	1
LOS	5.6 (±3.6)	14.6 (±12.4)	0.001
Morbidity	0 (0%)	28 (53.8%)	0.001
Mortality	2 (1.6%)	4 (7.7%)	0.05

SMI: skeletal muscle index BMI: body mass index; Charlson Comorbidity index; LOS: length of stay.

**Table 4:** Comparison of Low SMI versus Intermediate SMI versus Normal SMI.

Variables	Low SMI (31)	Intermediate SMI (79)	Normal SMI (127)	*p*
Age (y/o)	72 (±12.7)	63 (±14.5)	57 (±15.5)	<0.001
Gender				
Male	12 (39%)	30 (38%)	90 (71%)	<0.001
Female	19 (61%)	49 (62%)	37 (29%)	
BMI	23.3 (±3.4)	25.3 (±4.0)	26.5 (±4.7)	0.001
Frailty Index	2.2 (±1.2)	1.6 (±0.8)	1.5 (±0.8)	<0.001
CCI	3.6 (±2.7)	2.3 (±2.2)	1.9 (±2.5)	0.004
LOS	10.4 (±6.4)	9.4 (±8.0)	9.9 (±11.5)	0.88
Hinchey				0.6
2	22 (71%)	65 (82.2%)	103 (81.1%)	
3	3 (9.7%)	7 (8.9%)	11 (8.7%)	
4	6 (19.3%)	7 (8.9%)	13 (10.2%)	
Morbidity	11 (35.5%)	23 (29%)	27 (21%)	0.19
Anastomotic Leak	0 (0%)	1 (1.3%)	2 (1.6%)	0.78
Mortality	4 (12.9%)	0 (0%)	8 (6.3%)	0.014
Surgery	20 (64.5%)	42 (53.2%)	54 (42.5%)	0.059
NOM failure	7 (38.9%)	19 (33.9%)	26 (26.3%)	0.83
Type of surgery				<0.05
Hartmann	16 (80%)	24 (57.1%)	24 (44.4%)	
Sigmoidectomy	4 (20%)	16 (38.1%)	28 (51.9%)	
Other	0	2 (4.8%)	2 (3.7%)	

**Table 5:** Comparison of before 2020 treatment versus after 2020 treatment.

Variables	Before 2020 (193)	After 2020 (44)	*p*
NOM	139 (72%)	34 (77%)	0.5
NOM Failure	44 (22.8%)	8 (18.2%)	0.47
Surgery	98 (50.8%)	18 (40.9%)	0.24
Type of surgery			
Hartmann	57 (58.2%)	10 (55.5%)	0.84
Sigmoidectomy with anastomosis	26 (26.5%)	7 (38.9%)	0.28
Other	15 (15.3%)	1 (5.6%)	0.27

**Table 6:** Comparison between sarcopenic patients versus non-sarcopenic patients.

Variables	Not Sarcopenic (128)	Sarcopenic (109)	Univariate *p*-Value	Multivariate *p*-Value
Age	56.7 (±15.5)	66 (±14.4)	0.0001	0.32
Gender				
Male	91 (71%)	41 (37.6%)	0.0001	0.001
Female	37 (29%)	68 (62.4%)		
BMI	26.5 (±4.7)	24.7 (±3.9)	0.002	0.014
Hinchey 2	104 (81.3%)	86 (78.9%)	0.65	0.58
Hinchey 3	11 (8.6%)	10 (9.2%)	0.87	
Hinchey 4	13 (10.2%)	13 (11.9%)	0.66	
CCI	1.9 (±2.5)	2.6 (± 2.4)	0.03	0.12
Frailty Index	1.8 (±0.76)	2.4 (±0.87)	0.0001	0.09
Surgery first	28 (21.9%)	36 (33%)	0.056	
NOM	100 (78%)	73 (67%)	0.05	
NOM failure	26 (20.3%)	26 (23.8%)	0.5	0.81
LOS	9.9 (±11.5)	9.7 (±7.6)	0.8	
Mortality	8 (6.2%)	4 (3.7%)	0.5	

NOM: Non-operative management; LOS: length of stay.

**Table 7:** Comparison of sarcopenic versus non-sarcopenic patients among surgical population.

Variables	Not Sarcopenic (54)	Sarcopenic (62)	*p*
Age	60.5 (±16.2)	66.5 (±13.4)	0.03
Gender			
Male	38 (70.4%)	26 (41.9%)	0.002
Female	16 (29.6%)	36 (58.1%)	
BMI	25.7 (±3.5)	24.4 (±3.8)	0.05
LOS	16 (±15.4)	12.5 (±8.3)	0.12
Hinchey ≥ 3	20 (37.0%)	23 (37.1)	0.86
Surgery first	28 (52%)	36 (58%)	0.5
NOM failure	26 (48%)	26 (42%)	0.5
Type of surgery			
Resection and anastomosis	23 (42.6%)	14 (22.6%)	0.02
Hartmann’s Procedures	24 (44.4%)	40 (64.5%)	0.03
Ileostomy	5 (9.3%)	6 (9.7%)	0.94
Lavage and drain	2 (3.7%)	2 (3.2%)	0.89
Morbidity	28 (52%)	27 (43.5%)	0.37
Clavien-Dindo			
>2	18 (64.3%)	15 (55.6%)	0.5
Mortality	6 (11.1%)	3 (4.8%)	0.2

SMI: skeletal muscle index BMI: body mass index; Charlson Comorbidity index; LOS: length of stay; NOM: non-operative management.

**Table 8:** Comparison of sarcopenic versus non-sarcopenic patients among Hartmann’s procedure and resection and anastomosis.

Hartmann’s Procedure (64)				Resection and Anastomosis (37)		
	Not Sarcopenic (24)	Sarcopenic (40)	*p*	Not Sarcopenic (23)	Sarcopenic (14)	*p*
LOS	16.4 ± 16.2	13.5 ± 9.3	0.36	12.6 ± 10.4	10.2 ± 6.6	0.44
Hinchey						
2	11 (45.8%)	21 (52.5%)	0.3	20 (86.9%)	12 (85.7%)	0.21
3	3 (12.5%)	8 (20%)	0.44	2 (8.7%)	2 (14.3%)	0.59
4	10 (41.7%)	11 (27.5%)	0.24	1 (4.4%)	0	0.87
Morbidity	14 (58.3%)	13 (32.5%)	0.14	7 (30.4%)	4 (28.6%)	0.55
CD < 3a	11 (78.5%)	7 (53.9%)	0.17	4 (57%)	3 (75%)	0.55
CD ≥ 3a	3 (21.5%)	6 (46.1%)	0.17	3 (43%)	1 (25%)	0.55
Mortality	3 (12.5%)	8 (20%)	0.44	2 (8.7%)	0 (0%)	0.57

LOS: length of stay.

**Table 9:** Logistic regression model.

	*B*	*S.E.*	*Wald*	*Gl*	*Sign.*	*Exp(B)*	*C.I. Inf 95%*	*C.I. Sup 95%*
Sarcopenia	0.12	0.42	0.08	1	0.78	1.13	0.49	2.56
Age	0.00	0.02	0.001	1	0.98	1.00	0.97	1.03
Gender	−0.04	0.43	0.009	1	0.92	0.96	0.42	2.21
BMI	0.06	0.06	1.09	1	0.29	0.94	0.85	1.05
Frailty Score	−0.57	0.51	1.24	1	0.27	0.57	0.21	1.54
CCI	0.29	0.19	2.23	1	0.13	1.34	0.91	1.98
Costante	1.72	1.89	0.82	1	0.36	5.56		

## Data Availability

Data will be available after a specific reasonable request to the corresponding author.
